# Evaluation of Single-Impact-Induced Cartilage Degeneration by Optical Coherence Tomography

**DOI:** 10.1155/2015/486794

**Published:** 2015-07-01

**Authors:** Florence de Bont, Nicolai Brill, Robert Schmitt, Markus Tingart, Björn Rath, Thomas Pufe, Holger Jahr, Sven Nebelung

**Affiliations:** ^1^Department of Orthopaedics, Aachen University Hospital, 52074 Aachen, Germany; ^2^Fraunhofer Institute for Production Technology, Aachen, Germany; ^3^Laboratory for Machine Tools and Production Engineering, RWTH Aachen University, 52074 Aachen, Germany; ^4^Institute of Anatomy and Cell Biology, RWTH Aachen University, 52074 Aachen, Germany

## Abstract

Posttraumatic osteoarthritis constitutes a major cause of disability in our increasingly elderly population. Unfortunately, current imaging modalities are too insensitive to detect early degenerative changes of this disease. Optical coherence tomography (OCT) is a promising nondestructive imaging technique that allows surface and subsurface imaging of cartilage, at near-histological resolution, and is principally applicable* in vivo* during arthroscopy. Thirty-four macroscopically normal human cartilage-bone samples obtained from total joint replacements were subjected to standardized single impacts* in vitro* (range: 0.25 J to 0.98 J). 3D OCT measurements of impact area and adjacent tissue were performed prior to impaction, directly after impaction, and 1, 4, and 8 days later. OCT images were assessed qualitatively (DJD classification) and quantitatively using established parameters (*OII, Optical Irregularity Index; OHI, Optical Homogeneity Index; OAI, Optical Attenuation Index*) and compared to corresponding histological sections. While* OAI* and* OHI* scores were not significantly changed in response to low- or moderate-impact energies, high-impact energies significantly increased mean DJD grades (histology and OCT) and* OII* scores. In conclusion, OCT-based parameterization and quantification are able to reliably detect loss of cartilage surface integrity after high-energy traumatic insults and hold potential to be used for clinical screening of early osteoarthritis.

## 1. Introduction

Despite significant research efforts in the last decades and an ever-increasing understanding of the disease, osteoarthritis (OA) remains the most important pathology in orthopaedics, especially in ageing Western societies [1]. Next to other extrinsic factors like obesity and joint loading patterns, supraphysiological impact loading of articular cartilage is suggested to result in posttraumatic (i.e., secondary) OA [2]. In particular, posttraumatic OA may be the result of fractures, dislocations, ligament tears, or soft-tissue injuries and subsequent surface incongruity, instability, or altered joint kinematics [3]. Since novel therapies addressing cartilage degeneration in early stages of OA are available, the early noninvasive diagnosis of cartilage degradation is of utmost clinical interest [4]. Therefore, novel scientific and clinical imaging strategies are increasingly aiming at the detection of preosteoarthritic conditions and early cartilage degeneration. Conventional clinical imaging modalities for the assessment of cartilage such as X-ray, Magnetic Resonance Imaging (MRI), and arthroscopy lack the ability to reliably detect early degenerative changes: moderate-to-severe cartilage degeneration may be indirectly detected by conventional X-ray [5], while current clinical morphological MRI is limited in resolution and marked by deficits in interobserver reliability [6, 7]. Arthroscopy as an invasive imaging modality is considered the diagnostic gold standard [8]; yet it merely allows subjective surface evaluation and tactile probing of cartilage. The diagnosis of subsurface lesions is challenging, while differentiation between healthy and very early degenerative cartilage is thus virtually impossible [9–11]. Arthroscopic evaluation thus regularly fails to detect signs of early degeneration as evident after histological evaluation [12].

For this reason a true clinical need exists for an adjunct diagnostic tool that is capable of reliably identifying early degenerative changes in cartilage. Optical coherence tomography (OCT) is a promising imaging modality that may meet those demands. As a noninvasive imaging technique, analogous to ultrasound, it captures cross-sectional images of semitransparent tissues by detecting the interference of near-infrared light, thereby producing high-resolution images similar to low power histology [12, 13]. More specifically, comparative evaluation has revealed a high degree of congruence between OCT-based and histological imaging* in vitro* [12, 13] as well as* in vivo* [14, 15]. Principal* in vivo* applicability of OCT-based cartilage assessment has been demonstrated during arthroscopy and open knee surgery alike [14–16]. Recently, a number of quantitative optical and structural OCT parameters have been devised to objectively and reliably assess cartilage degeneration like fibrillation [12], reflection [17], signal scattering [18], and OCT signal intensities [11] as well as the combination of irregularity, homogeneity, and attenuation [13]. While first* ex vivo* data suggest a potential diagnostic value in the bovine system [11], OCT-based parameters in posttraumatic human cartilage degeneration have not yet been comprehensively evaluated in a standardized traumatic impact model. Literature data indicate that excessive injurious single-impact loading causes acute cartilage damage with increasing chondrocyte death and extracellular matrix damage. The initiated degenerative response subsequently predisposes to the clinical manifestation of posttraumatic OA in the long term [19]. For experimental studies, the drop-tower device is a well-established model to induce posttraumatic degenerative changes [20, 21]; likewise,* in vitro* single-impact loading and the initiation of OA-like histological changes were found to be well correlated [22].

Therefore, the objective of this study was to evaluate the diagnostic value of a set of quantitative OCT parameters in the assessment of posttraumatic human cartilage degeneration. Our hypothesis was that posttraumatic degeneration of human cartilage may be properly evaluated by quantitative OCT.

## 2. Materials and Methods

### 2.1. Preparation of Cartilage Samples

**Figure 1:**
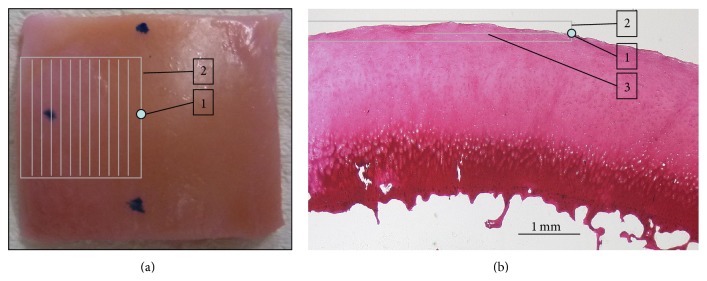
(a) Top view of a representative cartilage sample to illustrate the standardized scan area. Tissue marking dye spots at the 12, 6, and 9 o'clock positions indicate the midsagittal plane (between 12 and 6 o'clock) and its perpendicular plane, while the intersection point between both planes is the sample centre point (1; white spot). From here, 3D OCT scanning was performed parallel to the midsagittal plane in an area of 10 × 10 mm (length × width) towards the sample periphery as indicated by 9 o'clock dot (2; parallel lines within box). Thus, the impact area itself (3; centered on the centre point) and the immediate adjacent concentric tissue were scanned. (b) Corresponding histological section demonstrating the topography of the sample centre point (1) and the scan area (2) in relation to the radius of the impact area (3). Note the absence of subchondral cancellous bone which had been removed to leave only compact bone attached. Safranin O, 1.6x magnification.

Upon informed patient consent and institutional ethical review board approval (AZ EK 157/13), human articular cartilage-bone samples were obtained from total knee replacement surgeries (*n* = 11 patients; 7 males, 4 females; age 72.0 ± 5.0 years). All patients underwent total knee replacement at our institution due to primary OA of the knee as determined both clinically and radiographically. After sterile excision, cartilage-bone samples were collected in sterile DMEM medium (Gibco-BRL, Gaithersburg, USA) containing 100 U/mL penicillin (Gibco), 100 *μ*g/mL gentamycin (Gibco), and 1.25 U/mL amphotericin B (Gibco) and immediately transferred to the laboratory. Macroscopically, samples were graded according to the Outerbridge classification [23]. For standardization, only cartilage samples graded Outerbridge grade 0, that is, without any signs of degeneration, were used for the present study. Moreover, only samples obtained from the femoral condyles (9/25 [medial/lateral]) were included. Samples were cut to standard size (length × width: 20 × 20 mm) with the surface as plain as possible, while the subchondral bone was trimmed to the subchondral lamella; that is, all cancellous bone was removed until only compact bone was left. It is of note that the thickness of the subchondral bone plate as determined on histological sections was variable ranging from 0.10 to 0.75 mm. Tissue marking dye (Polysciences, Warrington, USA) outside the impact area was applied for future reference. More specifically, two spots at opposing sample sides marked the midsagittal imaging plane (0°), while its orthogonal plane (90°) was defined by a third spot on another sample side (Figure 1). The intersection of these two planes indicated the sample center point. Thirty-four samples were thus prepared and assessed macroscopically, of which 32 were included in the impaction versus nonimpaction study design (see Section 2.2) and thus transferred to 12-well plates filled with 3 mL/well of the DMEM medium as above. Two samples underwent histological processing immediately after preparation for preimpact histological standardization.

### 2.2. Standardized Impaction of Cartilage Samples

Samples were assigned to 4 groups consisting of 8 samples each; one nonimpacted control group and three groups receiving impacts of different energy levels (low impact, 0.25 J [LIMP]; moderate impact, 0.49 J [MIMP]; high impact, 0.98 J [HIMP]). A custom-made drop tower has been reported earlier [24] and shown to deliver standardized impacts. Its specifications (height 33 cm; diameter 4 cm; material polymethyl-methacrylate) allowed the dropping of iron cylinder weights contained within. Dropping defined cylindric weights (500 g; 1000 g) with a 5-mm diameter impactor tip from defined heights delivered three different energy levels to the cartilage surface (Table 1). Here, the drop height (*h*) determines the velocity of impact (*ν*) by *ν* = (2*gh*)^1/2^, where *g* is the acceleration because of gravity and height (*h*) and mass (*m*) together determine the energy by *E* = *mgh*. The different heights and masses were used to create “mild” (LIMP) to “severe” (HIMP) damage in cartilage. The area of impact was marked by tissue marking dye (Figure 1). After impact, the impactor tip was left on the well-wetted samples for 5 s to ensure constant compression conditions [22]. Subsequently, samples were cultured under standardized conditions (37°C; 5% CO_2_; humid air) in DMEM/additives-medium, which was changed every two days.

### 2.3. OCT-Based Imaging, Processing, and Assessment

The OCT device has already been reported previously [13, 25, 26]. Briefly, a spectral-domain OCT device (Thorlabs, Dachau, Germany) with a 1325 nm superluminescent diode (bandwidth: 150 nm; axial resolution: 7.5 *μ*m in air; lateral resolution: 15.6 *μ*m) was used. A CMOS camera in line with the OCT beam was used to guide OCT image acquisition. Before scanning, focusing, centration, and artefact minimization was performed to optimize imaging. Samples were scanned along the midsagittal plane using a galvoscanner setup. The region of interest was scanned in a standardized way to include the impact area itself and the immediate adjacent concentric area; more specifically, the sample centre point (i.e., centre of impact area) was included but provided the centre edge of the scan area which extended 10 mm to the periphery as defined by the peripheral edge of the scan area (Figure 1). Thus, samples were measured in an area of 10 × 10 × 2.5 mm [length × width × depth] corresponding to a 3D data matrix of 512 × 512 × 512 pixels [length × width × depth]. It is of note that 512 individual, successive 2D B-scans at 0.02 mm intervals were obtained for each 3D dataset. At an A-scan line imaging speed of 28 kHz, data acquisition was completed after a total of 9.4 sec per sample. For assessment of time-related changes, longitudinal OCT measurements took place at five different time points: prior to impaction to determine baseline characteristics (“preimpact”), immediately after impaction (“postimpact”) and after 1 (“day 1”), 4 (“day 4”), and 8 days (“day 8”), respectively. At each time point and after completion of OCT measurements two samples of each group underwent histological processing as outlined below for comparative evaluation.

Qualitative OCT scoring was performed according to the Degenerative Joint Disease (DJD) classification [27] by two blinded observers experienced in musculoskeletal histopathology (FDB, SN). Briefly, DJD grade 0 shows no structural alteration, while DJD grade 1 exhibits some surface irregularities such as wrinkling and fraying. Superficial cleft formation is seen in DJD grade 2, while deeper clefts extending to the transitional, radial, or tidemark zones are assigned DJD grades 3, 4, or 5, respectively. DJD grade 6 shows complete tissue disorganization or fibrous tissue replacement. Quantitative OCT image analysis was performed by custom software routines in LabVIEW Vision (National Instruments, Austin, USA) and Matlab (Mathworks Inc., Natick, USA). OCT parameters previously defined and validated in the context of primary human cartilage degeneration were used [13, 26]: surface irregularity (*OII*, Optical Irregularity Index), tissue homogeneity (*OHI*, Optical Homogeneity Index), and signal attenuation (*OAI*, Optical Attenuation Index). As before, image processing steps included artifact reduction, morphological filtering, and transformation of grey scale images to binary data. Individual A-scans were processed and the first pixel unequal to zero was detected. Thus, the actual surface topography outline was detected in individual B-scans and referenced to a smoothed surface dataset as obtained by Savitzky-Golay filtering.* OII* as a measure of surface integrity was then calculated as the standard deviation between the actual and the smoothed surfaces. Higher absolute* OII* values indicate higher surface irregularity and were demonstrated to be associated with more severe degeneration [13].* OHI *as a measure of inner-tissue homogeneity was determined by detection and quantification of signal contrast changes in individual A-scans. Higher absolute* OHI* values, that is, higher numbers of signal edges, indicate less tissue homogeneity and were equally demonstrated to be associated with more severe degeneration [13]. Imaging depth and corresponding attenuation were assessed by detecting the depth-wise signal propagation in individual A-scans.* OAI *was determined as the tissue depth where a signal intensity loss below 15% of the initial signal intensity at the sample surface was noted. Higher* OAI* values indicate less optical signal attenuation through the tissue, although no degeneration-dependent* OAI* increase was noted in contrast to* OII* and* OHI* [13].

It is of note that single OCT images matching the corresponding histological sections as below were evaluated to allow for intermethod comparison within the region of interest. As 512 OCT B-scan images were obtained of the scanned area of 10 × 10 mm (length × width), OCT images numbered 1, 102, 204, 306, 408, and 510 were evaluated to match the histological sectioning procedure.

### 2.4. Histological Analyses

Histological processing and evaluation have been reported earlier [13]. Briefly, samples were fixed and decalcified in Ossa fixona (Diagonal, Muenster, Germany) and sectioned along the midsagittal plane as outlined by the two opposing tissue marks. The sample half containing the perpendicular third tissue mark was used for further histological processing. In total, five transverse 5 *μ*m sections parallel to the midsagittal plane were cut at 2 mm intervals and stained with hematoxylin/eosin and Safranin O. Overall, a total width of 10 mm was thus covered starting from the central point of the sample and extending 10 mm into the sample periphery. Microscopic images (Leica, Wetzlar, Germany) were taken of the stained sections and prepared using the Diskus software (Leica). Qualitative histological grading on six tissue sections per sample was again performed by two blinded observers using the Degenerative Joint Disease (DJD) classification (*DJD scores*). Likewise, histological sections were assessed qualitatively in terms of proteoglycan content according to the specific subscore published by Mankin et al. [28]. Briefly, normal proteoglycan content, that is, Safranin O staining intensities, was graded 0, while slight/moderate/severe reductions in Safranin O staining were graded 1/2/3, respectively (*PG scores*). Overall, means of individual scores as obtained throughout the sample region of interest were calculated and used for further analysis. It is of note that a longitudinal assessment of histological outcomes was not performed.

### 2.5. Statistical Analysis

GraphPad Prism Software (Version 5.0, San Diego, CA, USA) was used for statistical analyses and plotting. As a result of unequal group sizes, the Kruskal-Wallis test followed by Dunn's post hoc test for pairwise comparisons was used to assess statistical differences between groups (i.e., control, LIMP, MIMP, and HIMP) and time points (i.e., preimpact, postimpact, day 1, day 4, and day 8). Moreover, kappa scores were calculated for interobserver reliability. *p* values of *p* ≤ 0.05 were considered statistically significant and exact levels of significance are specified in the figure legends, with [ns] denoting nonsignificant differences. Kappa scores are presented as *κ* [95% CI], and data in tables as means ± standard deviations; sample size (M ± SD; *n*).

## 3. Results

**Figure 2:**
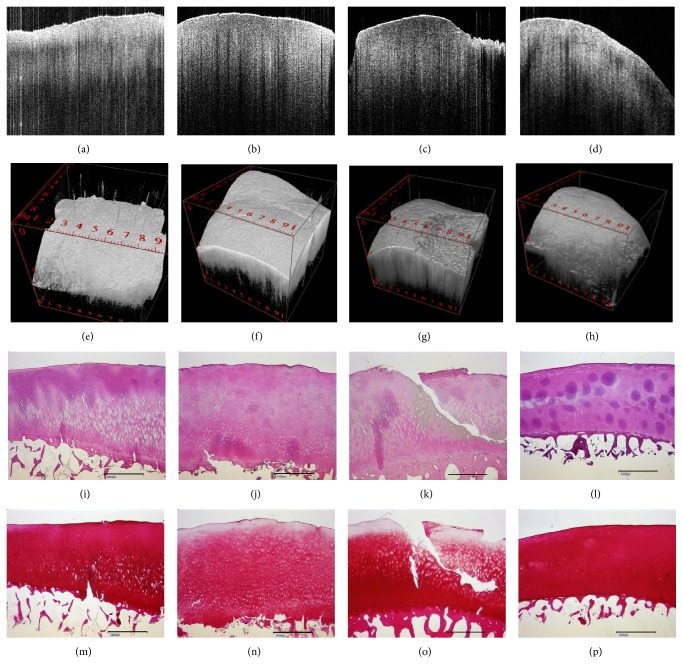
Correlation between OCT and histology. Impact-induced cartilage degeneration on top of corresponding representative 2D OCT B-scans (a–d), 3D OCT renderings (e–h), and histology images (i–l [HE]; m–p [Safranin O]); LIMP (a, e, i, m), MIMP (b, f, j, n), HIMP (c, g, k, o) postimpact, and controls (d, h, l, p). Bar represents 1 mm; OCT images fit to scale of histological sections, while entire image width in 3D OCT renderings represents 10 mm. For an explanation of the abbreviations please see Table 2.

**Figure 3:**
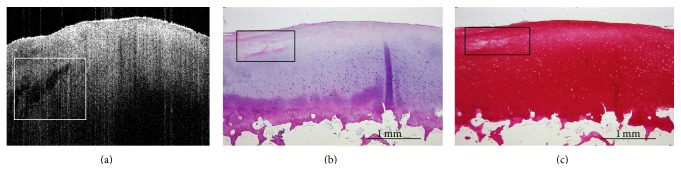
Subsurface changes (i.e., cleft formation) in response to single-impact loading. Exemplary sample (MIMP, day 8) with impact area on the left half of each image. Rectangles indicate close-to-surface fissures in the 2D OCT image (a) which were confirmed by histology: HE (b), Safranin O (c). OCT image fits to scale of histological sections.

**Figure 4:**
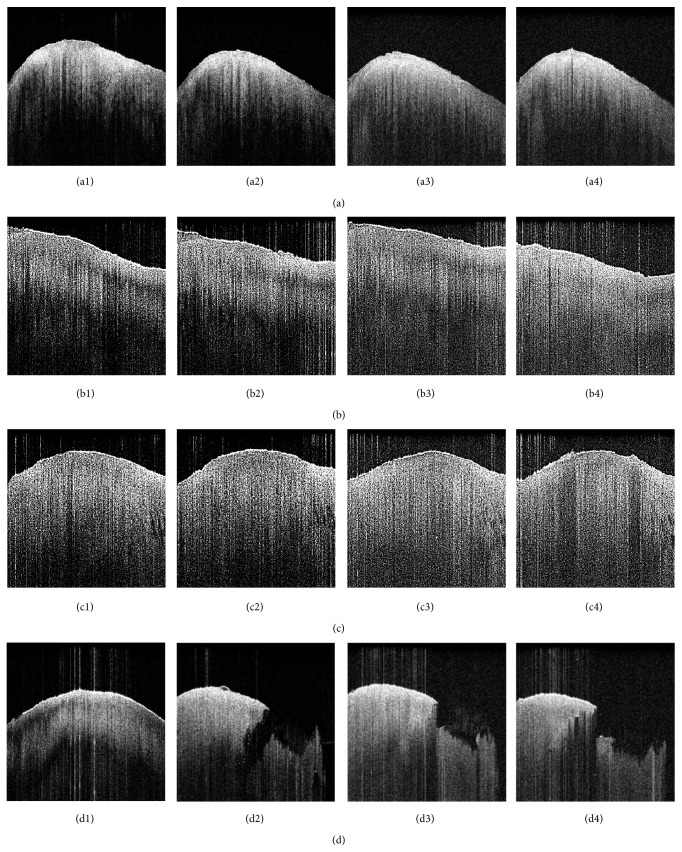
Example OCT images of time-related changes in response to single-impact loading; (a) controls; (b) LIMP; (c) MIMP; (d) HIMP. Preimpact (1); postimpact (2); day 4 (3); day 8 (4). Midsagittal images along sample centre point are displayed. Entire individual image width represents 10 mm.

Qualitative histological grading provided the reference standard for qualitative OCT-based assessment of matched OCT images. Overall, 72% of the samples (26/36) correlated well between OCT-based and histological DJD grading. Interobserver reliability for histology and OCT was found to be *κ*_Histology_ = 0.785 [CI = 0.628–0.942] and *κ*_OCT_ = 0.714 [CI = 0.538–0.890). Posttraumatic changes resulted in significantly different histological mean DJD and PG scores in response to high-impact loading (Table 2). Interestingly, intergroup assessment of PG content revealed differences (*p* = 0.046), while no such differences were found upon pairwise posttest comparisons (Table 2). Likewise, pairwise posttest comparisons revealed histological intergroup differences for DJD grades (*p* = 0.025), with increasing grades in the HIMP group as compared to controls (controls versus HIMP [*∗*]; Table 2). Correspondingly, intergroup differences were also found for DJD grades determined by OCT (*p* < 0.001) and pairwise posttest comparisons revealed increased DJD grades upon comparison of the HIMP group versus all other groups (controls versus HIMP [*∗∗*]; LIMP versus HIMP [*∗*]; MIMP versus HIMP [*∗*]; Table 2). In response to low- or moderate-impact loading no such changes were observed (Table 2). Qualitative assessment of image morphologies supports these findings with marked changes observed only in response to high-impact loading (Figure 2). Moreover, macroscopic examination revealed significant dents after high-impact loading exclusively, while no gross indentation marks were apparent in the other groups. Subsurface changes, for example, cleft formations, were detectable in OCT images which could be confirmed in the corresponding histological sections in response to all impact energy levels (Figure 3). Time-related qualitative assessment of cartilage revealed distinctive surface and subsurface changes in response to loading. In all groups, by trend, subsurface homogeneity was found to be increased and attenuation to be decreased in the course of cultivation, while surface irregularity was considerably increased in the HIMP group only (Figure 4). It is of note that surface peaks and valleys tended to be less prominent later on in the cultivation process.

*OII* was increased in response to high-impact loading with significant differences between preimpact and postimpact as well as day-1 measurements ((1),* OII*, HIMP, *p* < 0.001) (Tables 3 and 4). In contrast,* OII* did not change upon low- or moderate-impact loading in time either, while intergroup assessment revealed differences in* OII* values at the postimpact time point (*p* = 0.039). However, with pairwise post hoc testing the differences only remained significant between moderate- and high-impact loading (MIMP versus HIMP [*∗*]).

*OHI* time-dependently decreased in all groups, regardless of the magnitude of impaction, while group-wise comparisons at individual time points revealed no significant differences (Table 3; (2),* OHI*, controls, *p* < 0.001; (3),* OHI*, LIMP, *p* < 0.001; (4),* OHI*, MIMP, *p* = 0.003; (5),* OHI*, HIMP, *p* < 0.001).* OHI* values dropped significantly between directly postimpact data and day-1 measurements. The latter is supported by Dunn's post hoc testing revealing numerous differences between pre- and postimpact versus day-1 and subsequent measurements (Table 4).

Correspondingly, significant time-dependent increases were found for* OAI* following postimpact measurements in all groups (Table 3; (6),* OAI*, controls, *p* < 0.001; (7),* OAI*, LIMP, *p* = 0.002; (8),* OAI*, MIMP, *p* = 0.018; (9),* OAI*, HIMP, *p* < 0.001), while intergroup analysis revealed no significant differences. Conversely,* OAI* values were found to be significantly increased at day-1 measurements with a similar high number of differences being observed between pre- and postimpact versus day-1 and subsequent measurements (Table 4).

## 4. Discussion

The most important finding of the present study is the ability of OCT- and image-based parameterization and quantification to assess secondary degenerative changes of human articular cartilage.

Histological assessment of cartilage is the current gold standard in judging cartilage degeneration, but its practical clinical value is limited due to its invasiveness. Our results support previous studies indicating that OCT is principally applicable to detecting cartilage changes without removal of cartilage tissue [11, 13, 29]. In this study, OCT images were compared and correlated to matching histological sections in a traumatic cartilage degeneration model. Overall, 72% of the samples correlated well between OCT and histological assessment. Residual disagreements may have been due to several factors such as histological processing and subsequent artefact introduction as well as mismatching of OCT and histology images. Literature data indicate a strong correlation of* in vitro* single-impact loading and the initiation of OA-like changes [22]. The different impact energies used in this study are in line with previously reported impact energies [24, 30]. Different height-weight combinations allowed for a systematic evaluation of impact-induced changes. The histology data indicate that the applied impact energy level is closely related to the subsequently occurring changes, with higher impacts causing more severe changes. Overall, these findings were confirmed by reference histology focusing on cartilage structure (i.e., DJD grading) and composition (i.e., proteoglycan content). Our findings are generally in line with previous studies [24, 30, 31]. Cartilage has unique compressive and viscoelastic properties that serve the purpose of absorbing mechanical loads [32]. Impact loading to chondral explants resulted in cell death at levels as low as 3–6 MPa [31, 33], while in contrast others have suggested thresholds of 15 −20 MPa for triggering chondrocyte death [21, 34]. Cartilage function is believed to deteriorate as a result of chondrocyte death and changes in the biochemical composition of the extracellular matrix as well as in the biomechanical properties [35, 36]. Generally, cell death was found to increase with time and impact energy and to gradually spread to adjacent tissue areas [37].

A significant increase as compared to controls was found for histology and* OII* scores after high-energy impact exclusively, while low-to-moderate energy impact did not affect either parameter. This observation may be due to a threshold of energy impact required to initiate irreversible cartilage damage as demonstrated earlier [21, 30, 38]. More specifically, these studies indicate certain impact thresholds (in terms of strain rates, impact stresses, and energies) at which permanent changes to the structural and functional setup of cartilage become evident. In contrast, Duda et al. suggested that considerable damage is sustained at the cellular level before structural destruction of cartilage [31]. Furthermore, subtle damage to the matrix can occur even before the cartilage surface is damaged [31]. When using different energies ranging from 0.06 J to 0.2 J to traumatically impact cartilage, no gross macroscopic changes were found while chondrocyte viability was reduced. This finding is in line with our data which indicate no significant change in histological or OCT parameters in response to low- to moderate-energy impact (LIMP: 0.25 J; MIMP: 0.49 J) thereby supporting the threshold theory above.

In other studies, high impact resulted in early degenerative changes, while low impact exhibited a delayed biological response [39]. With close to 2 J of impact energy, severe fissures across the cartilage surface and throughout the depth of bovine articular cartilage were reported. In general, considerable decreases in the biomechanical properties may require impact energies larger than 1.5 J [30]; 2.8 J also triggered chondrocyte death and decreased biomechanical properties within 24 h, while 1.1 J caused little changes even within one week after impact [39]. Most studies tend to report impact measurements in various ways and may therefore be difficult to compare directly. Because of the nature of its viscoelastic response and because it is thin (generally only 2-3 mm), cartilage may be a poor shock absorber [40]. Leaving the bone attached to the samples had a strong protective effect and increasing the impact energy was found to result in damage to the bone rather than to the cartilage [24], an effect not investigated within the present study.

However, the present study found small subsurface changes such as tissue disruptions as a result of traumatic impaction that were essentially detectable by OCT and could be confirmed by corresponding histology. Although these local changes were clearly assessable on single 2D OCT B-scans in terms of location and extension, their effects on quantified parameters were limited due to data pooling within the 3D OCT dataset (consisting of 512 images). As demonstrated before [26], 3D OCT-based imaging and assessment of cartilage in terms of rendering, surface topography, and parametric and multiple cross-sectional views allow for more efficient areal tissue assessment (instead of arbitrary single-plane imaging of single joint sites); yet, local changes may have been missed by the image analysis algorithms used in the present study. Future work is necessary to implement algorithms for the automatic detection of subsurface tissue disruptions. It is of note that the tissue disruptions were found to run parallel to the articular surface, which may be due to deflection mechanisms within the transition zone of the extracellular matrix as hypothesized earlier [24].

Early stages of cartilage degeneration are associated with a loss of proteoglycans [41], collagen network integrity [42], and cartilage surface integrity [43]. In both primary and secondary cartilage degeneration the earliest alterations include surface irregularity formation, erosion, and fissuring and may be consistently detected by OCT.* OII* as a measure surface irregularity was the only OCT parameter to be significantly affected by impaction, that is, high-energy impact, whereas* OHI *and* OAI *were not.* OII* may therefore be considered a useful marker for structural changes, although very early degenerative changes (i.e., DJD grades 0 versus 1) may thus not be differentiated as demonstrated before [13]. Here, future studies that thoroughly investigate different surface profile parameters may potentially improve the diagnostic performance in very early degeneration. The simultaneous decrease of* OHI* and increase of* OAI* were independent of impaction.* In vitro* conditions differ from the* in vivo* situation within a joint [44] and tissue explantation may initiate degenerative processes during incubation that potentially increased signal propagation (i.e., higher* OAI*) and increased tissue homogeneity (i.e., lower* OHI*). Significant changes* in OHI* and* OAI* were first measurable after 24 h of cultivation.

Limitations of the present study include tissue quality and integrity which are important factors to consider. Although we exclusively used macroscopically intact human cartilage samples for this study, these were obtained from knees undergoing joint replacement surgery. As OA is a disease that affects the entire joint, even “intact” cartilage may have been subtly damaged on a microscopic level [45]. Chu et al. found early degenerative changes in healthy cartilage as assessed arthroscopically [12], while Spahn et al. found a substantial variability in differentiating between intact and degenerative cartilage [9]. Our longitudinal study design, including pre- and postmeasurements, may have reduced this bias, although its contribution to data variability cannot be entirely excluded. In particular, submicroscopic alteration of the specialized surface zone, with its horizontally orientated collagen fibrils, in predamaged samples (even though macroscopically unaffected) could have resulted in samples being more prone to rapid mechanical damage. Other limitations involve the relatively small sample size used in our study and the fact that impact load transmission and dissipation may have been affected by the status of the subchondral bone plate, which had been left attached (to better recreate the physiological situation). Among others, Madry et al. have elucidated the science of the subchondral bone plate and its changes in chronic OA [46]. Future studies should also take alternative read-out parameter of cartilage degeneration, like biomechanical and/or biochemical evaluation, into account. Further refined analyses algorithms may facilitate differential assessment of distinct tissue depths and areas in follow-up studies. The experimental setup of the present study, using osteochondral cores and focal impaction, may limit its translation to a clinically relevant whole-joint setting. As discussed above, most changes were observed in response to high-impact loading, which may indicate the presence of an impaction threshold. Previous work by Borrelli and Ricci (reviewed in [47]) demonstrated that defined traumatic impaction of osteochondral cores caused more severe postimpact changes than those observed in intact joints after similar impact regimes. Hence, this particular impaction threshold seems to be related to the overall tissue properties in terms of structure, composition, and geometry. As load dissipation characteristics in the intact joint undergoing a supraphysiological, traumatic impaction of cartilage surfaces are likely different from those in the present* ex vivo* study, our results may not be entirely transferable to the actual clinical setting.

## 5. Conclusion

Despite some technical limitations, the present study is the first to systematically assess overall tissue changes in response to traumatic impaction in a close-to-clinical setting by use of OCT- and image-based analysis algorithms. Above a certain threshold, impact-induced degenerative changes in cartilage may be successfully assessed by OCT. In particular, OCT-based parameterization and quantification seem to be of diagnostic benefit in detecting loss of surface integrity as a result of traumatic impaction.

## Figures and Tables

**Table 1:** Impact energy and velocities corresponding to masses and drop heights as described by Jeffrey et al. [24].

Mass [g]	Drop height [mm]	Energy [J]	Impact velocity [m/s]	Impact group
500	50	0.2	0.99	Low impact (LIMP)
500	100	0.49	1.40	Moderate impact (MIMP)
1000	100	0.98	1.40	High impact (HIMP)

**Table 2:** Summary of histology- and OCT-based grading of human cartilage degeneration following standardized impact with low (LIMP), moderate (MIMP), or high (HIMP) energy. Scoring of degenerative changes was performed according to the Degenerative Joint Disease (DJD) classification or the proteoglycan content subscore of the Mankin classification. Nonparametric Kruskal-Wallis test followed by Dunn's post hoc testing was performed for statistical analyses. Data are presented as M ± SD; *n*. Dunn's posttesting revealed controls versus HIMP (histological DJD) and all groups versus HIMP (OCT DJD) to be significantly different. It is of note that one sample in the control group was lost during the histological preparation (*n* = 9).

	Controls	LIMP	MIMP	HIMP	*p* value
Proteoglycan content (histology)	0.74 ± 0.51; 9	0.79 ± 0.43; 8	0.88 ± 0.7; 8	1.73 ± 0.91; 8	**0.046** [*∗*]
DJD grade (histology)	0.98 ± 0.59; 9	1.12 ± 0.49; 8	1.30 ± 1.06; 8	2.62 ± 1.3; 8	**0.025** [*∗*]
DJD grade (OCT)	0.73 ± 0.60; 9	1.02 ± 0.48; 8	1.00 ± 0.61; 8	2.65 ± 0.86; 8	**<0.001** [*∗∗∗*]

[*∗*]: 0.01 ≤ *p* ≤ 0.05; [*∗∗∗*]: *p* < 0.001.

**Table 3:** Quantitative OCT parameter values as a function of time (preimpaction; postimpaction; after 1 day; after 4 days; after 8 days) in response to three different impact energies. Data are presented as M ± SD; *n*; statistical differences were assessed using nonparametric Kruskal-Wallis test to assess differences between individual time points [tp] and groups [gr], respectively. Dunn's post hoc testing revealed significant differences (bold) primarily between time points (# 1–9), while differences between impact modalities were found for postimpact, *OII* only. Here, pairwise comparison revealed only MIMP versus HIMP to be different (*∗*). Consecutive numbers indicate posttest details as found in Table 4. Data are presented as M ± SD; *n*. Table legend is as in Table 2.

		Controls	LIMP	MIMP	HIMP	*p* value (gr)
*OII *	Preimpact	7.395 ± 2.684; 10	7.492 ± 1.762; 8	5.596 ± 2.200; 8	5.279 ± 1.496; 8	0.059
	Postimpact	7.660 ± 1.983; 10	7.817 ± 1.252; 8	8.099 ± 5.112; 8	10.14 ± 1.693; 8	**0.039** (*∗*)
	Day 1	7.567 ± 2.211; 8	7.836 ± 1.629; 6	6.613 ± 3.908; 6	9.788 ± 1.454; 6	0.088
	Day 4	6.739 ± 1.502; 6	7.861 ± 2.218; 4	6.708 ± 6.261; 4	8.814 ± 0.786; 4	0.274
	Day 8	6.721 ± 1.576; 4	7.040 ± 3.737; 2	4.644 ± 0.002; 2	9.496 ± 0.148; 2	0.400
	*p* value (tp)	0.846	0.992	0.903	**<0.001 (1)**	

*OHI *	Preimpact	2.190 ± 0.33; 10	2.110 ± 0.227; 8	2.121 ± 0.57; 8	2.224 ± 0.207; 8	0.873
	Postimpact	2.272 ± 0.419; 10	2.222 ± 0.27; 8	2.225 ± 0.505; 8	1.971 ± 0.251; 8	0.203
	Day 1	1.274 ± 0.288; 8	1.210 ± 0.218; 6	1.162 ± 0.341; 6	1.054 ± 0.116; 6	0.441
	Day 4	1.366 ± 0.322; 6	1.364 ± 0.121; 4	1.175 ± 0.305; 4	1.135 ± 0.199; 4	0.460
	Day 8	1.270 ± 0.298; 4	1.473 ± 0.104; 2	1.221 ± 0.300; 2	1.016 ± 0.044; 2	0.519
	*p* value (tp)	**<0.001 (2)**	**<0.001 (3)**	**0.003 (4)**	**<0.001 (5)**	

*OAI *	Preimpact	59.9 ± 27.6; 10	63.2 ± 33.4; 8	79.6 ± 68.4; 8	77.3 ± 19.0; 8	0.535
	Postimpact	67.2 ± 29.9; 10	69.8 ± 39.1; 8	81.3 ± 60.8; 8	69.2 ± 25.4; 8	0.985
	Day 1	149.7 ± 38.3; 8	177.3 ± 9.8; 6	172.5 ± 42.4; 6	164.1 ± 16.2; 6	0.461
	Day 4	168.0 ± 41.2; 6	159.5 ± 36.1; 4	192.2 ± 42.3; 4	155.4 ± 18.4; 4	0.517
	Day 8	151.8 ± 39.4; 4	132.2 ± 51.5; 2	199.2 ± 7.8; 2	155.5 ± 18.1; 2	0.454
	*p* value (tp)	**<0.001 (6)**	**0.002 (7)**	**0.018 (8)**	**<0.001 (9)**	

**Table 4:** Dunn's post hoc testing details as outlined by consecutive numbers found in Table 3. Significant differences are marked by asterisks ([*∗∗∗*]: *p* < 0.001; [*∗∗*]: 0.001 ≤ *p* ≤ 0.01; [*∗*]: 0.01 ≤ *p* ≤ 0.05; [ns]: nonsignificant). Table legend is as in Table 2.

	*OII* HIMP (1)	*OHI* controls (2)	*OHI* LIMP (3)	*OHI* MIMP (4)	*OHI* HIMP (5)	*OAI* controls (6)	*OAI* LIMP (7)	*OAI* MIMP (8)	*OAI* HIMP (9)
Preimpact versus postimpact	*∗∗*	ns	ns	ns	ns	ns	ns	ns	ns
Preimpact versus day 1	*∗*	*∗∗*	*∗*	ns	*∗∗*	*∗∗*	*∗∗*	ns	*∗*
Preimpact versus day 4	ns	*∗*	ns	ns	*∗*	*∗∗*	ns	ns	ns
Preimpact versus day 8	ns	*∗*	ns	ns	ns	*∗*	ns	ns	ns
Postimpact versus day 1	ns	*∗∗*	*∗∗*	*∗*	ns	*∗*	*∗*	ns	*∗∗*
Postimpact versus day 4	ns	*∗*	*∗*	ns	ns	*∗*	ns	ns	*∗*
Postimpact versus day 8	ns	*∗*	ns	ns	ns	ns	ns	ns	ns
Day 1 versus day 4	ns	ns	ns	ns	ns	ns	ns	ns	ns
Day 1 versus day 8	ns	ns	ns	ns	ns	ns	ns	ns	ns
Day 4 versus day 8	ns	ns	ns	ns	ns	ns	ns	ns	ns

## References

[1] Felson D. T., Zhang Y. (1998). An update on the epidemiology of knee and hip osteoarthritis with a view to prevention. *Arthritis and Rheumatism*.

[2] Chu C. R., Williams A. A., Coyle C. H., Bowers M. E. (2012). Early diagnosis to enable early treatment of pre-osteoarthritis. *Arthritis Research and Therapy*.

[3] Brown T. D., Johnston R. C., Saltzman C. L., Marsh J. L., Buckwalter J. A. (2006). Posttraumatic osteoarthritis: a first estimate of incidence, prevalence, and burden of disease. *Journal of Orthopaedic Trauma*.

[4] Lotz M. K. (2010). New developments in osteoarthritis. Posttraumatic osteoarthritis: pathogenesis and pharmacological treatment options. *Arthritis Research & Therapy*.

[5] Vignon E., Piperno M., Le Graverand M.-P. H. (2003). Measurement of radiographic joint space width in the tibiofemoral compartment of the osteoarthritic knee: comparison of standing anteroposterior and Lyon schuss views. *Arthritis and Rheumatism*.

[6] Krampla W., Roesel M., Svoboda K., Nachbagauer A., Gschwantler M., Hruby W. (2009). MRI of the knee: how do field strength and radiologist's experience influence diagnostic accuracy and interobserver correlation in assessing chondral and meniscal lesions and the integrity of the anterior cruciate ligament?. *European Radiology*.

[7] Saarakkala S., Laasanen M. S., Jurvelin J. S. (2003). Ultrasound indentation of normal and spontaneously degenerated bovine articular cartilage. *Osteoarthritis and Cartilage*.

[8] Oakley S. P., Portek I., Szomor Z. (2005). Arthroscopy—a potential ‘gold standard’ for the diagnosis of the chondropathy of early osteoarthritis. *Osteoarthritis and Cartilage*.

[9] Spahn G., Klinger H. M., Baums M., Pinkepank U., Hofmann G. O. (2011). Reliability in arthroscopic grading of cartilage lesions: results of a prospective blinded study for evaluation of inter-observer reliability. *Archives of Orthopaedic and Trauma Surgery*.

[10] Spahn G., Klinger H. M., Hofmann G. O. (2009). How valid is the arthroscopic diagnosis of cartilage lesions? Results of an opinion survey among highly experienced arthroscopic surgeons. *Archives of Orthopaedic and Trauma Surgery*.

[11] Bear D. M., Szczodry M., Kramer S., Coyle C. H., Smolinski P., Chu C. R. (2010). Optical coherence tomography detection of subclinical traumatic cartilage injury. *Journal of Orthopaedic Trauma*.

[12] Chu C. R., Lin D., Geisler J. L., Chu C. T., Fu F. H., Pan Y. (2004). Arthroscopic microscopy of articular cartilage using optical coherence tomography. *The American Journal of Sports Medicine*.

[13] Nebelung S., Marx U., Brill N. (2014). Morphometric grading of osteoarthritis by optical coherence tomography—an ex vivo study. *Journal of Orthopaedic Research*.

[14] Chu C. R., Izzo N. J., Irrgang J. J., Ferretti M., Studer R. K. (2007). Clinical diagnosis of potentially treatable early articular cartilage degeneration using optical coherence tomography. *Journal of Biomedical Optics*.

[15] Chu C. R., Williams A., Tolliver D., Kwoh C. K., Bruno S., Irrgang J. J. (2010). Clinical optical coherence tomography of early articular cartilage degeneration in patients with degenerative meniscal tears. *Arthritis and Rheumatism*.

[16] Li X., Martin S., Pitris C. (2005). High-resolution optical coherence tomographic imaging of osteoarthritic cartilage during open knee surgery. *Arthritis Research & Therapy*.

[17] Saarakkala S., Wang S.-Z., Huang Y.-P., Zheng Y.-P. (2009). Quantification of the optical surface reflection and surface roughness of articular cartilage using optical coherence tomography. *Physics in Medicine and Biology*.

[18] Han C. W., Chu C. R., Adachi N. (2003). Analysis of rabbit articular cartilage repair after chondrocyte implantation using optical coherence tomography. *Osteoarthritis and Cartilage*.

[19] D'Lima D., Hermida J., Hashimoto S., Colwell C., Lotz M. (2006). Caspase inhibitors reduce severity of cartilage lesions in experimental osteoarthritis. *Arthritis and Rheumatism*.

[20] Repo R. U., Finlay J. B. (1977). Survival of articular cartilage after controlled impact. *The Journal of Bone and Joint Surgery—American Volume*.

[21] Torzilli P. A., Grigiene R., Borrelli J., Helfet D. L. (1999). Effect of impact load on articular cartilage: cell metabolism and viability, and matrix water content. *Journal of Biomechanical Engineering*.

[22] Huser C. A. M., Davis M. E. (2006). Validation of an in vitro single-impact load model of the initiation of osteoarthritis-like changes in articular cartilage. *Journal of Orthopaedic Research*.

[23] Outerbridge R. E. (1961). The etiology of chondromalacia patellae. *The Journal of Bone & Joint Surgery—British Volume*.

[24] Jeffrey J. E., Gregory D. W., Aspden R. M. (1995). Matrix damage and chondrocyte viability following a single impact load on articular cartilage. *Archives of Biochemistry and Biophysics*.

[25] Marx U., Schmitt R., Nebelung S., Tingart M., Luring C., Rath B., Vo-Dinh T., Mahadevan-Jansen A., Grundfest W. In vitro observation of cartilage-degeneration progression by Fourier-domain OCT.

[26] Nebelung S., Brill N., Marx U. (2015). Three-dimensional imaging and analysis of human cartilage degeneration using Optical Coherence Tomography. *Journal of Orthopaedic Research*.

[27] Xie T., Guo S., Zhang J., Chen Z., Peavy G. M. (2006). Determination of characteristics of degenerative joint disease using optical coherence tomography and polarization sensitive optical coherence tomography. *Lasers in Surgery and Medicine*.

[28] Mankin H. J., Dorfman H., Lippiello L., Zarins A. (1971). Biochemical and metabolic abnormalities in articular cartilage from osteo-arthritic human hips. II. Correlation of morphology with biochemical and metabolic data. *The Journal of Bone and Joint Surgery—American Volume*.

[29] Virén T., Huang Y. P., Saarakkala S. (2012). Comparison of ultrasound and optical coherence tomography techniques for evaluation of integrity of spontaneously repaired horse cartilage. *Journal of Medical Engineering & Technology*.

[30] Verteramo A., Seedhom B. B. (2007). Effect of a single impact loading on the structure and mechanical properties of articular cartilage. *Journal of Biomechanics*.

[31] Duda G. N., Eilers M., Loh L., Hoffman J. E., Kääb M., Schaser K. (2001). Chondrocyte death precedes structural damage in blunt impact trauma. *Clinical Orthopaedics and Related Research*.

[32] Mansour J., Oatis C. A. (2004). Biomechanics of cartilage. *Kinesiology: The Mechanics and Pathomechanics of Human Movement*.

[33] Fehrenbacher A., Steck E., Rickert M., Roth W., Richter W. (2003). Rapid regulation of collagen but not metalloproteinase 1, 3, 13, 14 and tissue inhibitor of metalloproteinase 1, 2, 3 expression in response to mechanical loading of cartilage explants in vitro. *Archives of Biochemistry and Biophysics*.

[34] Milentijevic D., Torzilli P. A. (2005). Influence of stress rate on water loss, matrix deformation and chondrocyte viability in impacted articular cartilage. *Journal of Biomechanics*.

[35] Blanco F. J., Guitian R., Vázquez-Martul E., de Toro F. J., Galdo F. (1998). Osteoarthritis chondrocytes die by apoptosis. A possible pathway for osteoarthritis pathology. *Arthritis and Rheumatism*.

[36] Malemud C. J., Shuckett R., Goldberg V. M. (1988). Changes in proteoglycans of human osteoarthritic cartilage maintained in explant culture: implications for understanding repair in osteoarthritis. *Scandinavian Journal of Rheumatology, Supplement*.

[37] Levin A., Burton-Wurster N., Chen C.-T., Lust G. (2001). Intercellular signalling as a cause of cell death in cyclically impacted cartilage explants. *Osteoarthritis and Cartilage*.

[38] Quinn T. M., Allen R. G., Schalet B. J., Perumbuli P., Hunziker E. B. (2001). Matrix and cell injury due to sub-impact loading of adult bovine articular cartilage explants: effects of strain rate and peak stress. *Journal of Orthopaedic Research*.

[39] Natoli R. M., Scott C. C., Athanasiou K. A. (2008). Temporal effects of impact on articular cartilage cell death, gene expression, matrix biochemistry, and biomechanics. *Annals of Biomedical Engineering*.

[40] Radin E. L., Paul I. L., Lowy M. (1970). A comparison of the dynamic force transmitting properties of subchondral bone and articular cartilage. *The Journal of Bone & Joint Surgery—American Volume*.

[41] van de Loo A. A. J., Arntz O. J., Otterness I. G., van den Berg W. B. (1994). Proteoglycan loss and subsequent replenishment in articular cartilage after a mild arthritic insult by IL-1 in mice: impaired proteoglycan turnover in the recovery phase. *Agents and Actions*.

[42] Hamerman D., Klagsbrun M. (1985). Osteoarthritis. Emerging evidence for cell interactions in the breakdown and remodeling of cartilage. *American Journal of Medicine*.

[43] Pritzker K. P. H., Gay S., Jimenez S. A. (2006). Osteoarthritis cartilage histopathology: grading and staging. *Osteoarthritis and Cartilage*.

[44] Nebelung S., Gavenis K., Lüring C. (2012). Simultaneous anabolic and catabolic responses of human chondrocytes seeded in collagen hydrogels to long-term continuous dynamic compression. *Annals of Anatomy*.

[45] Kleeman R. U., Krocker D., Cedrano A., Tuischer J., Duda G. N. (2005). Altered cartilage mechanics and histology in knee osteoarthritis: relation to clinical assessment (ICRS Grade). *Osteoarthritis and Cartilage*.

[46] Madry H., van Dijk C. N., Mueller-Gerbl M. (2010). The basic science of the subchondral bone. *Knee Surgery, Sports Traumatology, Arthroscopy*.

[47] Borrelli J., Ricci W. M. (2004). Acute effects of cartilage impact. *Clinical Orthopaedics and Related Research*.

